# Robot-assisted thoracoscopic lobectomy as treatment of a giant bulla

**DOI:** 10.1186/s13019-017-0595-3

**Published:** 2017-05-18

**Authors:** Rosa Roemers, Kornelis Patberg, Caroline van de Wauwer, Tam Nguyen, Ghada Shahin

**Affiliations:** 1 Department of Cardio-thoracic Surgery, Universitair Medisch Centrum Groningen (UMCG), Hanzeplein 1, 9700 RB Groningen, The Netherlands; 2 Department of Cardio-thoracic Surgery, Isala Clinics Zwolle, Dokter van Heesweg 2, 8025 AB Zwolle, The Netherlands; 3 Department of Pulmonology, Isala Clinics Zwolle, Dokter van Heesweg 2, 8025 AB Zwolle, The Netherlands

**Keywords:** Giant Congenital Bulla, Bullectomy, Robot-assisted thoracoscopic lobectomy, Case-report

## Abstract

**Background:**

A bulla is a marked enlarged space within the parenchyma of the lung. Bullae may cause dyspnea by compressing healthy lung parenchyma and can cause a pneumothorax. Also, bullae are associated with malignancy, therefore surgical bullectomy is indicated on preventive basis. This case is unique and therefore valuable because of the remarkable presentation, innovative treatment and the spectacular improvement of lung function and socio-economic performance of the patient.

**Case presentation:**

In this case report we describe the presentation, minimally invasive surgical treatment by means of a robot-assisted lobectomy and postoperative outcome of a young patient with a giant congenital bulla of the left upper lobe.

**Conclusions:**

In this case robot-assisted lobectomy has shown spectacular improvement of lung function and fast-track recovery with beneficial socio-economic performance in a young patient with a giant congenital bulla.

## Background

A bulla is a marked enlarged space within the parenchyma of the lung with thinning and destructed alveolar septa; it has a diameter greater than one centimeter in the distended state. If the bulla occupies at least 30% of a hemithorax it is considered a giant bulla [1]. A bulla may reach a considerable size and occasionally ruptures leading to pneumothorax [1, 2]. Mediastinal displacement and compressive effects of the affected lung can be caused by a large bulla. Many patients with giant bullae are asymptomatic but symptoms might include dyspnea due to compression of otherwise healthy parenchym, palpitations, chest pain or recurrent infections; however, when discovered treatment is imperative since these bullae may be associated with malignancy or in time degenerate into a malignancy. This was described by a number of case reports and series in which bronchogenic carcinoma developed within or adjacent to a giant bulla [3–7]. Diagnosis is made on chest radiography and CT scanning whereas detection of any associated malignancy requires more profound investigation especially in case of diffuse tumor growth. Radiographic patterns suggestive of a possible malignant process include nodular opacities within or next to the bulla, partial or multi focal thickening of the wall of the bulla, and secondary signs of the bulla such as pneumothorax, fluctuating diameter and fluid retention [8].

Endobronchial stenting with one-way endobronchial valves to deflate the bulla has been described [9]. However, based on the association with malignancy (as just described) and the sheer size of the bulla we decided to treat this patient surgically. Surgical bullectomy is indicated on preventive basis when a patient is diagnosed with a giant bulla, a growing bulla or a bulla that is compressing healthy adjacent lung tissue [1]. Surgical bullectomy can be performed via an open thoractomy or via a thoracoscopic approach, depending on patient specific factors. In this case we describe the use of the Da Vinci Si™ surgical robot for the thoracoscopic approach of the bullectomy.

## Case presentation

A 15-year old girl was referred to an orthopedic surgeon with complaints of a consistent nagging pain in her left shoulder which were provoked by physical exertion and had persisted for over a year. Shoulder radiography revealed no abnormalities of the left shoulder joint, although a remarkable abnormal aspect of the left upper part of the chest was described, for which she was referred to the Pulmonology Department.

Additional patient history revealed limited physical exercise tolerance compared to her peers, mostly due to dyspnea and pain; she had no other complaints. She had no history of smoking or drug abuse. Her medical history was unremarkable except for a fall from a horse one year prior to presentation. The family history was negative for lung disease. Physical examination showed a female adolescent with diminished breath sounds over the left upper part of the chest. Oxygen saturation was normal. There were no other abnormal clinical findings.

**Fig. 1:**
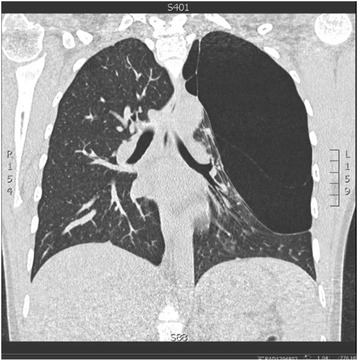
Pre-operative high resolution CT scan, showing the massive bulla (17 × 12 cm) of the *left* upper lobe with compression of the remaining lung parenchyma

Spirometry showed a vital capacity of 3.18 L (85%), FEV1 2.79 l (84%) and a total lung capacity of 5.04 (111%). *High Resolution CT scanning* of the chest showed a massive bulla (17 × 12 cm) of the left upper lobe (LUL) with compression of the residual lung parenchyma (Fig. 1). There were no signs of underlying pulmonary disease or other congenital pathology. Laboratory tests were all within the normal range including the alpha-1-antitrypsin level.

### Surgical procedure

It was decided to perform a robot-assisted thoracoscopic (RATS) lobectomy to resect the left upper lobe in this young patient, in order to reduce hyperinflation, and improve exercise capacity and exercise tolerance. Simultaneously this would reduce the risk of associated malignacy or rupture of the bulla resulting in a pneumothorax.

Surgery was performed under general anesthesia with double lumen endotracheal intubation and single lung ventilation. The usual antibiotic profylaxis was administered, amoxicillin/clavulanic acid 1000/200 mg intravenously. The patient was positioned in right lateral position, port placement and docking of the Da Vinci Si surgical robot (Intuitive Surgical Inc., Sunnyvale CA, USA) took place using the 4-arms technique. This technique is described extensively by Cerfolio et al. [10]. The left upper lobe (LUL) was dominated by giant bulla with only a small amount of normal parenchyma present. The left lower lobe was intact without any bullae. We took down the apical adhesions and as the bulla was impairing our vision it was opened to create space. All the arteries to the LUL were transected using vascular loads for the Johnson&Johnson Endopath Powered Echelon 45 mm stapling device. Although we dissected on the central artery there was an unusually large amount of very small arteries supplying the LUL (probably indicative of the minimal perfusion of the affected lobe). After identification of both pulmonary veins the left upper pulmonary vein was transected using the above-mentioned stapler. The left upper lobe bronchus was divided after test clamping and inflation of the lower lobe, using a bronchial load for the stapling device. Lastly, several lymph nodes were dissected and sent for pathological examination. The specimen was removed in an endobag and the chest cavity drained with one chest tube. For pain management, we instilled Chirocaine para vertebral block at five levels with additional local infiltration of the port sites. The RATS procedure was accomplished without mentionable blood loss or other intraoperative complications.

### Histopathological examination

**Fig. 2:**
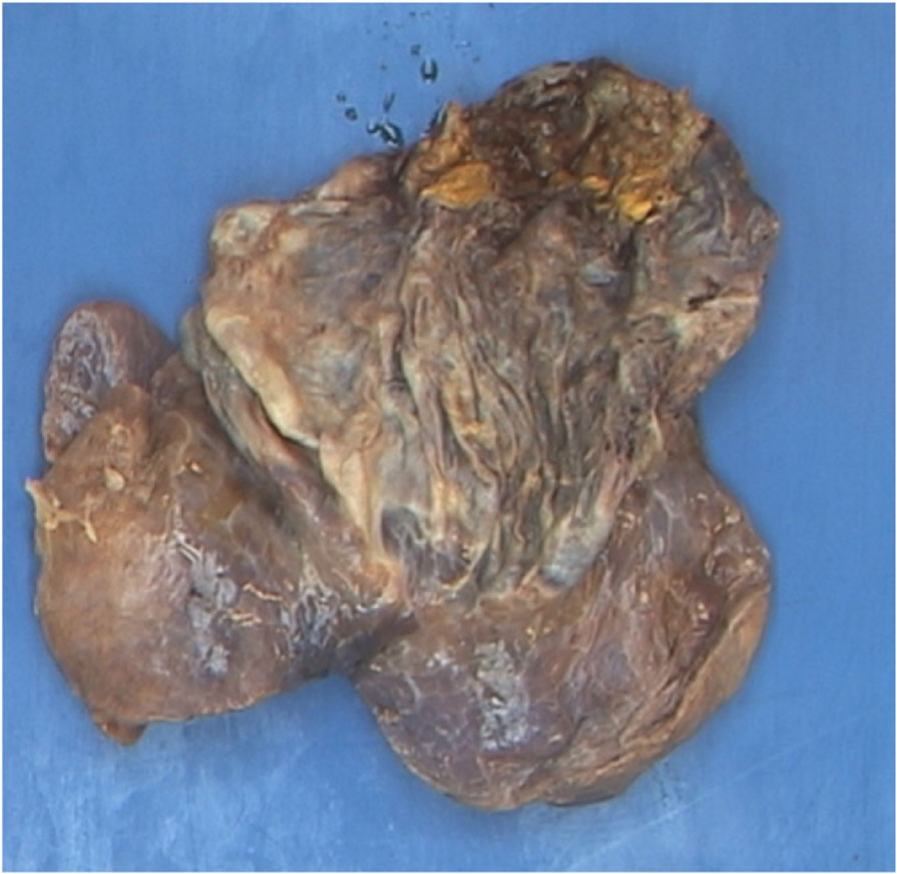
Resected giant bulla preserved with formaldehyde fluid. Weight of 250 g, 17 × 12 cm

On histopathological examination, the lymph nodes showed no abnormalities. Examination of the lining of the cystic cavity showed collagenous stroma, prominent capillaries and a few lymphoid aggregates. There were a few multinucleated giant cell macrophages on the surface, but there was no coating of ciliated epithelium. There was no cartilage found in the cyst wall. Furthermore, there was no coating of bronchial epithelium and therefore not enough arguments for the diagnosis congenital pulmonary airway malformation and all histopathological findings indicate a giant bulla [11–13] (Fig. 2).

### Postoperative findings

Our patient had an uneventful postoperative course. The chest tube was removed on the second postoperative day. On the third day after surgery she was discharged in good clinical condition. Routine chest X-Ray before discharge showed a small persistent pneumothorax due to the inability of the left lower lobe to fill up the entire left chest. On follow-up two and a half months later the patient was in good clinical condition with a remarkable decrease of dyspnea sensation. The chest X-Ray showed no pneumothorax, just a minor elevation of the left hemi-diaphragm, which is a normal post-operative finding after this procedure. Follow-up spirometry showed a reduction of total lung capacity from 5.04 L to 4.20 L. The residual volume had spectacularly decreased from 1.87 L (192% of predicted) to 0.78 L (80% of predicted). RV/TLC decreased as well from 157% of predicted to 79% of predicted.

## Discussion and Conclusions

We present a case of a young patient with a giant congenital bulla that was successfully resected with RATS lobectomy. There are no randomized controlled trials that support the use of Video Assisted Thoracoscopic Surgery (VATS), let alone RATS, over the traditional thoracotomy to perform a bullectomy. However, in the last decade minimal invasive surgery with endoscopic staple resection of bulla has shown to be an appropriate and safe treatment [8, 14].

The advantages of RATS over VATS in general are the 3D HD vision and the fact that the console surgeon is his own camera man. The dexterity and precision of surgery that are made possible by the Endowrist technology of the instruments cannot be surpassed by the VATS technique in which the surgeon works with rigid instruments. Finally, in our experience our patients recover faster and better with less pain than our VATS patients.

The biggest (current) disadvantage of the RATS approach is the price. However preliminary data from our group shows improved quality of life after RATS compared to VATS.

By performing the lobectomy through a minimally invasive technique we spared this 15-year-old girl a thoracotomy. Her hospital stay was short, she could resume school within three days postoperative. The fast recovery and impressive improvement of lung function is important, especially in this age group. We argue that a giant bulla is a rare but harmful lesion, therefore we advocate early detection and active treatment. In our opinion patients with giant bulla should receive surgical resection; resection not only improves mechanical properties of respiration but also prevents potential acute complications such as pneumothorax, and potentially associated occult lung malignancy can be detected in an early stage [3–7].

## Abbreviations

LUL: Left upper lobe
RATS: Robot assisted thoracoscopic surgery
VATS: Video assisted thoracoscopic surgery
